# Methyl 2-(3-chloro­benzamido)­benzoate

**DOI:** 10.1107/S1600536812046934

**Published:** 2012-11-24

**Authors:** Abdelaaziz Ouahrouch, Moha Taourirte, Hassan B. Lazrek, Mohamed El Azhari, Mohamed Saadi, Lahcen El Ammari

**Affiliations:** a Laboratoire de Chimie Bio-organique et Macromoléculaire, Faculté des Sciences et Techniques Guéliz, Marrakech, Morocco; b Unité de Chimie Biomoléculaire et Médicinale, Faculté des Sciences Semlalia, Marrakech, Morocco; c Laboratoire de la Matière Condensée et des Nanostructures, Faculté des Sciences et Techniques Guéliz, Marrakech, Morocco; d Laboratoire de Chimie du Solide Appliquée, Faculté des Sciences, Université Mohammed V-Agdal, Avenue Ibn Battouta, BP 1014, Rabat, Morocco

## Abstract

In the mol­ecule of the title compound, C_15_H_12_ClNO_3_, the chloro­benzamide and benzoate units are almost co-planar, with a dihedral angle between the six-membered rings of 2.99 (10)°. An intra­molecular N—H⋯O hydrogen bond occurs. In the crystal, each mol­ecule is linked to a symmetry-equivalent counterpart across a twofold rotation axis by weak C—H⋯O and C—H⋯Cl hydrogen bonds, forming dimers. The packing is stabilized through weak π–π stacking along the *b-*axis direction, leading to π-stacked columns of inversion-related mol­ecules, with an inter­planar distance of 3.46 (2) Å and a centroid–centroid vector of 3.897 (2) Å.

## Related literature

For details of the synthesis, see: Shariat & Abdollahi (2004 ▶); Xingwen *et al.* (2007 ▶); Chandrika *et al.* (2008 ▶). For background to the potential biological use of benzoxazinone derivatives, see: Kurosaki & Naishi (1983 ▶); Ponchet *et al.* (1988 ▶); Hedsrom *et al.* (1984 ▶); Krantz *et al.* (1990 ▶).
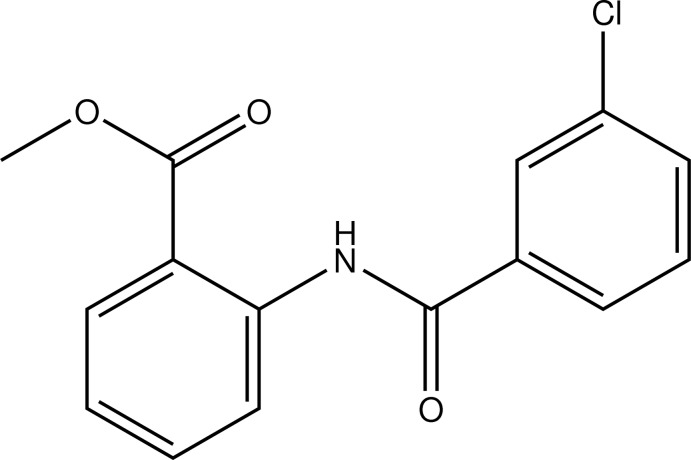


## Experimental

### 

#### Crystal data

C_15_H_12_ClNO_3_
*M* *_r_* = 289.71
Monoclinic, 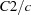

*a* = 25.7464 (10) Å
*b* = 6.9203 (2) Å
*c* = 16.9735 (6) Å
β = 116.045 (2)°
*V* = 2717.10 (16) Å^3^
*Z* = 8
Mo *K*α radiation
μ = 0.29 mm^−1^
*T* = 296 K
0.36 × 0.31 × 0.27 mm

#### Data collection

Bruker X8 APEXII diffractometer
Absorption correction: multi-scan (*SADABS*; Bruker, 2009 ▶) *T*_min_ = 0.957, *T*_max_ = 0.997
20126 measured reflections
3511 independent reflections
2143 reflections with *I* > 2σ(*I*)
*R*_int_ = 0.038

#### Refinement

*R*[*F* ^2^ > 2σ(*F* ^2^)] = 0.047
*wR*(*F* ^2^) = 0.144
*S* = 1.02
3511 reflections
181 parameters
H-atom parameters constrained
Δρ_max_ = 0.31 e Å^−3^
Δρ_min_ = −0.40 e Å^−3^

### 

Data collection: *APEX2* (Bruker, 2009 ▶); cell refinement: *SAINT* (Bruker, 2009 ▶); data reduction: *SAINT*; program(s) used to solve structure: *SHELXS97* (Sheldrick, 2008 ▶); program(s) used to refine structure: *SHELXL97* (Sheldrick, 2008 ▶); molecular graphics: *ORTEP-3 for Windows* (Farrugia, 2012 ▶) and *Mercury* (Macrae *et al.* 2008 ▶); software used to prepare material for publication: *PLATON* (Spek, 2009 ▶) and *publCIF* (Westrip, 2010 ▶).

## Supplementary Material

Crystal structure: contains datablock(s) I, global. DOI: 10.1107/S1600536812046934/zl2515sup1.cif

Structure factors: contains datablock(s) I. DOI: 10.1107/S1600536812046934/zl2515Isup2.hkl

Supplementary material file. DOI: 10.1107/S1600536812046934/zl2515Isup3.cml

Additional supplementary materials:  crystallographic information; 3D view; checkCIF report

## Figures and Tables

**Table 1:** Hydrogen-bond geometry (Å, °)

*D*—H⋯*A*	*D*—H	H⋯*A*	*D*⋯*A*	*D*—H⋯*A*
N1—H2⋯O2	0.86	1.96	2.6506 (19)	137
C1—H1⋯O1^i^	0.93	2.67	3.573 (2)	163
C9—H9⋯Cl1^i^	0.93	2.87	3.617 (2)	139

Symmetry code: (i) 
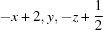
.

**Fig. 1.**
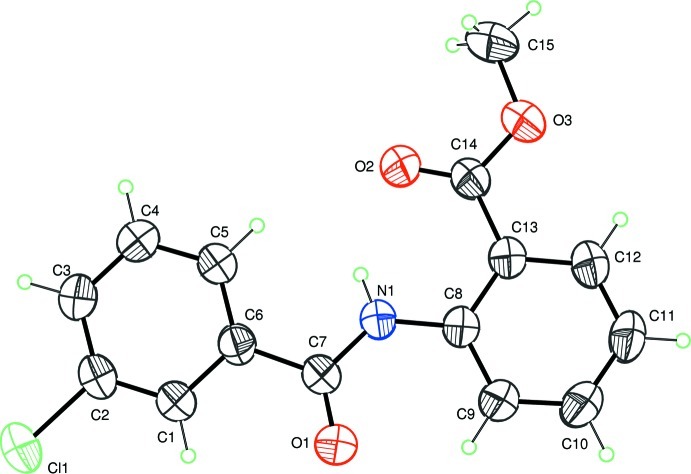
Molecular structure of the title compound with the atom-labelling scheme. Displacement ellipsoids are drawn at the 50% probability level. H atoms are represented as small circles.

**Fig. 2.**
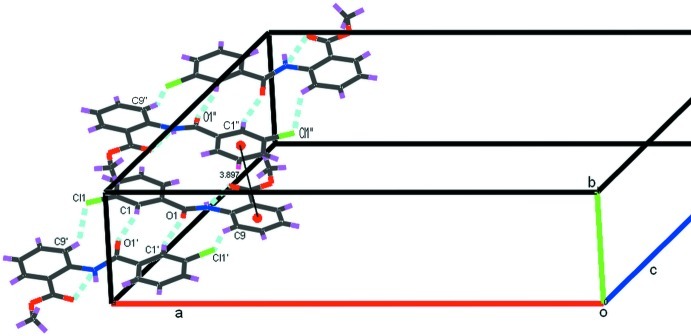
Packing of molecules through hydrogen bonds and π-stacking. Symmetry codes:(') -x+2, y, -z+1/2; (") -x+2, -y+1, -z+1.

## supplementary crystallographic information

### Comment

Benzoxazinone derivatives were found to be inhibitors of standard serine proteases of the chrymotrypsin superfamily (Kurosaki & Naishi, 1983, Ponchet *et al.*, 1988) and inhibit by formation of an acyl-enzyme complex through attack of the active site serine on the carbonyl group (Hedsrom *et al.*, 1984, Krantz *et al.*, 1990). The benzoxazinone derivatives have two available sites for nucleophilic attack. The two sites have partial positive charges that can guide different types of nucleophiles towards the opening of the heterocyles of the benzoxazinone derivatives. In general, benzoxazinone derivatives show good reactivity towards substitution reactions at position number 2 of the heterocycle and at positions 5, 6, 7 and 8 of the aromatic ring (Shariat & Abdollahi 2004). In connection to our studies on the synthesis of new bis-hyterocyclic compounds with different substituents, we decided to attempt to open the benzoxazinone heterocycle under basic conditions. Ring opening of 2-(3-chlorophenyl)-benzo[d][1,3] oxazin-4-one with potassium carbonate in methanol yielded the title compound.

The crystal structure of the methyl-2-(3-chlorobenzamido)benzoate features two aromatic six-membered rings (C1 to C6 and C8 to C13) which are, as expected, virtually planar, with maximum deviations of 0.006 (2) Å and -0.004 (2) Å for C1 and C9, respectively as shown in Fig.1. Moreover, the two rings are nearly coplanar as indicated by the dihedral angle between them of 2.99 (10) °. The two rings are linked through a connecting amide group with a C6—C7—N1—C8 dihedral angle of 174.71 (17)° (anti-periplanar conformation).

The cohesion of the molecules in the crystal structure is ensured by C1—H1···O1 and C9—H9···Cl1 non classic weak hydrogen bonds between symmetry equivalent molecules across a twofold rotation axis, forming dimers (Fig.2 and Table 2, symmetry operator (i) -x+2, y, -z+1/2). The structure is further stabilized by weak π–π stacking interactions between inversion-related molecules (symmetry operator (ii) -x+2, -y+1, -z+1), with an interplanar distance of 3.46 (2) Å and a centroid–centroid vector of 3.897 (2) Å, leading to formation of π-stacked columns of inversion-related molecules along the direction of the b-axis.

### Experimental

In the first step, follwowing a literature procedure (Xingwen *et al.*, 2007; Chandrika *et al.*, 2008) anthranilic acid (2-amino benzoic acid) was reacted with benzoyl chloride in dry pyridine at 273 K for 4 h to obtain 2-(3-chlorophenyl)-benzo[*d*][1,3] oxazin-4-one in good yield (75%). This products was then mixed with 0.5 eq of potassium carbonate in methanol to form the title compound in close to quantitative yield. The crude product was purified by passing through a column packed with silica gel. The solvent used for column chromatography was methylene chloride. A colourless crystal suitable for X-ray analysis was obtained by slow evaporation of a solution in methanol. The compound was characterized by ^1^H and ^13^C NMR and its structure was confirmed by X-ray diffraction analysis. ^1^H NMR (300 MHz, CDCl_3_) δ (ppm): 3.95 (s, 3H, -OCH_3_), 7.08-7.14 (m, 1H, H-Aromatic), 7.40-7.61 (m, 3H, H-Aromatic), 7.86 (d, 1H, H-Aromatic), 8.02-8.07 (m, 2H, H-Aromatic), 8.85 (m, 1H, H-Aromatic), 11.90 (s, 1H, -NH-). ^13^C NMR (300 MHz, CDCl_3_) δ (ppm): 52.54 (-OCH_3_), 120.47, 122.89, 125.10, 128.05, 130.05, 130.98, 131.94, 134.84 (CH-Aromatic), 115.28, 135.10, 136.71, 141.56 (C-Aromatic), 164.45 (CO), 169.05 (CO).

### Refinement

H atoms were located in a difference map and treated as riding with C—H = 0.96 Å, C—H = 0.97 Å, C—H = 0.93 Å and N—H = 0.86 Å for methyl, methylene, aromatic CH and NH respectively. All hydrogen atoms were refined with *U*_iso_(H) = 1.2 *U*_eq_ (aromatic, methylene) or *U*_iso_(H) = 1.5 *U*_eq_ for methyl.

### Crystal data

**Table:**

C_15_H_12_ClNO_3_	*F*(000) = 1200
*M**_r_* = 289.71	*D*_x_ = 1.416 Mg m^−^^3^
Monoclinic, *C*2/*c*	Melting point: 361.5 K
Hall symbol: -c 2yc	Mo *K*α radiation, λ = 0.71073 Å
*a* = 25.7464 (10) Å	Cell parameters from 3511 reflections
*b* = 6.9203 (2) Å	θ = 3.1–28.7°
*c* = 16.9735 (6) Å	µ = 0.29 mm^−^^1^
β = 116.045 (2)°	*T* = 296 K
*V* = 2717.10 (16) Å^3^	Block, colourless
*Z* = 8	0.36 × 0.31 × 0.27 mm

### Data collection

**Table:**

Bruker X8 APEXII diffractometer	3511 independent reflections
Radiation source: fine-focus sealed tube	2143 reflections with *I* > 2σ(*I*)
Graphite monochromator	*R*_int_ = 0.038
φ and ω scans	θ_max_ = 28.7°, θ_min_ = 3.1°
Absorption correction: multi-scan (*SADABS*; Bruker, 2009)	*h* = −34→34
*T*_min_ = 0.957, *T*_max_ = 0.997	*k* = −6→9
20126 measured reflections	*l* = −22→22

### Refinement

**Table:**

Refinement on *F*^2^	Primary atom site location: structure-invariant direct methods
Least-squares matrix: full	Secondary atom site location: difference Fourier map
*R*[*F*^2^ > 2σ(*F*^2^)] = 0.047	Hydrogen site location: inferred from neighbouring sites
*wR*(*F*^2^) = 0.144	H-atom parameters constrained
*S* = 1.02	*w* = 1/[σ^2^(*F*_o_^2^) + (0.0663*P*)^2^ + 1.0088*P*] where *P* = (*F*_o_^2^ + 2*F*_c_^2^)/3
3511 reflections	(Δ/σ)_max_ = 0.001
181 parameters	Δρ_max_ = 0.31 e Å^−^^3^
0 restraints	Δρ_min_ = −0.40 e Å^−^^3^

### Special details

**Table:**

Geometry. All e.s.d.'s (except the e.s.d. in the dihedral angle between two l.s. planes) are estimated using the full covariance matrix. The cell e.s.d.'s are taken into account individually in the estimation of e.s.d.'s in distances, angles and torsion angles; correlations between e.s.d.'s in cell parameters are only used when they are defined by crystal symmetry. An approximate (isotropic) treatment of cell e.s.d.'s is used for estimating e.s.d.'s involving l.s. planes.
Refinement. Refinement of *F*^2^ against all reflections. The weighted *R*-factor *wR* and goodness of fit *S* are based on *F*^2^, conventional *R*-factors *R* are based on *F*, with *F* set to zero for negative *F*^2^. The threshold expression of *F*^2^ > σ(*F*^2^) is used only for calculating *R*-factors(gt) *etc*. and is not relevant to the choice of reflections for refinement. *R*-factors based on *F*^2^ are statistically about twice as large as those based on *F*, and *R*- factors based on all data will be even larger.

### Fractional atomic coordinates and isotropic or equivalent isotropic displacement parameters (Å^2^)

**Table:**

	*x*	*y*	*z*	*U*_iso_*/*U*_eq_	
C1	1.07779 (8)	0.3043 (3)	0.40699 (12)	0.0493 (4)	
H1	1.0601	0.3084	0.3460	0.059*	
C2	1.13694 (8)	0.3154 (3)	0.45261 (13)	0.0532 (5)	
C3	1.16464 (8)	0.3122 (3)	0.54250 (13)	0.0576 (5)	
H3	1.2047	0.3199	0.5720	0.069*	
C4	1.13189 (9)	0.2973 (3)	0.58835 (13)	0.0598 (5)	
H4	1.1500	0.2962	0.6493	0.072*	
C5	1.07250 (8)	0.2840 (3)	0.54427 (12)	0.0523 (5)	
H5	1.0508	0.2732	0.5758	0.063*	
C6	1.04473 (7)	0.2867 (2)	0.45306 (11)	0.0450 (4)	
C7	0.98059 (8)	0.2750 (3)	0.40006 (12)	0.0491 (4)	
C8	0.89076 (8)	0.2154 (3)	0.41741 (12)	0.0479 (4)	
C9	0.85177 (9)	0.2244 (3)	0.32896 (13)	0.0643 (6)	
H9	0.8653	0.2354	0.2865	0.077*	
C10	0.79331 (9)	0.2169 (4)	0.30448 (14)	0.0770 (7)	
H10	0.7677	0.2245	0.2453	0.092*	
C11	0.77171 (9)	0.1984 (4)	0.36551 (15)	0.0751 (7)	
H11	0.7321	0.1932	0.3479	0.090*	
C12	0.80976 (8)	0.1878 (3)	0.45261 (14)	0.0613 (5)	
H12	0.7955	0.1743	0.4940	0.074*	
C13	0.86918 (8)	0.1966 (3)	0.48050 (11)	0.0470 (4)	
C14	0.90870 (8)	0.1895 (3)	0.57552 (12)	0.0497 (4)	
C15	0.91577 (12)	0.1812 (5)	0.71856 (14)	0.0935 (9)	
H15A	0.8921	0.1509	0.7476	0.112*	
H15B	0.9333	0.3057	0.7375	0.112*	
H15C	0.9454	0.0851	0.7327	0.112*	
N1	0.95046 (6)	0.2249 (2)	0.44555 (10)	0.0496 (4)	
H2	0.9712	0.1946	0.4996	0.060*	
O1	0.95798 (6)	0.3078 (3)	0.32160 (9)	0.0813 (5)	
O2	0.96083 (6)	0.1874 (2)	0.60722 (8)	0.0627 (4)	
O3	0.88031 (6)	0.1841 (2)	0.62469 (9)	0.0710 (4)	
Cl1	1.17831 (2)	0.33637 (11)	0.39517 (4)	0.0870 (3)	

### Atomic displacement parameters (Å^2^)

**Table:**

	*U*^11^	*U*^22^	*U*^33^	*U*^12^	*U*^13^	*U*^23^
C1	0.0453 (10)	0.0606 (11)	0.0458 (10)	−0.0014 (8)	0.0234 (8)	−0.0021 (8)
C2	0.0452 (10)	0.0637 (12)	0.0583 (11)	−0.0011 (8)	0.0298 (9)	−0.0003 (9)
C3	0.0397 (10)	0.0745 (13)	0.0562 (11)	−0.0021 (9)	0.0190 (9)	−0.0015 (9)
C4	0.0485 (11)	0.0805 (14)	0.0461 (11)	−0.0045 (10)	0.0169 (9)	−0.0017 (9)
C5	0.0454 (11)	0.0680 (12)	0.0473 (10)	−0.0040 (9)	0.0238 (9)	−0.0012 (8)
C6	0.0406 (9)	0.0493 (9)	0.0479 (10)	−0.0006 (7)	0.0219 (8)	−0.0016 (8)
C7	0.0439 (10)	0.0626 (11)	0.0445 (10)	−0.0023 (8)	0.0228 (8)	−0.0027 (8)
C8	0.0378 (9)	0.0596 (11)	0.0458 (10)	−0.0027 (8)	0.0178 (8)	−0.0009 (8)
C9	0.0446 (11)	0.1000 (16)	0.0459 (11)	−0.0092 (11)	0.0175 (9)	−0.0012 (10)
C10	0.0443 (12)	0.123 (2)	0.0511 (12)	−0.0117 (12)	0.0092 (10)	0.0035 (12)
C11	0.0351 (10)	0.1150 (19)	0.0692 (14)	−0.0091 (11)	0.0173 (10)	0.0055 (13)
C12	0.0434 (11)	0.0823 (14)	0.0628 (12)	−0.0034 (10)	0.0276 (10)	0.0032 (11)
C13	0.0405 (10)	0.0532 (10)	0.0479 (10)	−0.0017 (8)	0.0198 (8)	−0.0001 (8)
C14	0.0472 (11)	0.0575 (11)	0.0495 (10)	−0.0008 (8)	0.0259 (9)	0.0026 (8)
C15	0.0829 (18)	0.157 (3)	0.0497 (13)	0.0046 (17)	0.0375 (13)	0.0061 (14)
N1	0.0364 (8)	0.0701 (10)	0.0430 (8)	0.0001 (7)	0.0180 (7)	0.0044 (7)
O1	0.0493 (9)	0.1499 (15)	0.0442 (8)	−0.0092 (9)	0.0199 (7)	0.0100 (8)
O2	0.0461 (8)	0.0951 (11)	0.0464 (7)	−0.0013 (7)	0.0198 (6)	0.0058 (7)
O3	0.0586 (9)	0.1105 (12)	0.0529 (8)	0.0034 (8)	0.0326 (7)	0.0034 (7)
Cl1	0.0521 (3)	0.1486 (7)	0.0740 (4)	−0.0057 (3)	0.0405 (3)	0.0009 (4)

### Geometric parameters (Å, º)

**Table:**

C1—C2	1.374 (3)	C9—C10	1.375 (3)
C1—C6	1.391 (2)	C9—H9	0.9300
C1—H1	0.9300	C10—C11	1.380 (3)
C2—C3	1.372 (3)	C10—H10	0.9300
C2—Cl1	1.7374 (18)	C11—C12	1.371 (3)
C3—C4	1.380 (3)	C11—H11	0.9300
C3—H3	0.9300	C12—C13	1.390 (3)
C4—C5	1.379 (3)	C12—H12	0.9300
C4—H4	0.9300	C13—C14	1.482 (3)
C5—C6	1.392 (3)	C14—O2	1.207 (2)
C5—H5	0.9300	C14—O3	1.329 (2)
C6—C7	1.496 (3)	C15—O3	1.448 (3)
C7—O1	1.218 (2)	C15—H15A	0.9600
C7—N1	1.358 (2)	C15—H15B	0.9600
C8—C9	1.394 (3)	C15—H15C	0.9600
C8—N1	1.397 (2)	N1—H2	0.8600
C8—C13	1.412 (2)		
			
C2—C1—C6	119.22 (17)	C8—C9—H9	120.0
C2—C1—H1	120.4	C9—C10—C11	121.7 (2)
C6—C1—H1	120.4	C9—C10—H10	119.2
C3—C2—C1	122.16 (17)	C11—C10—H10	119.2
C3—C2—Cl1	118.55 (15)	C12—C11—C10	118.80 (19)
C1—C2—Cl1	119.28 (15)	C12—C11—H11	120.6
C2—C3—C4	118.71 (18)	C10—C11—H11	120.6
C2—C3—H3	120.6	C11—C12—C13	121.57 (19)
C4—C3—H3	120.6	C11—C12—H12	119.2
C5—C4—C3	120.37 (18)	C13—C12—H12	119.2
C5—C4—H4	119.8	C12—C13—C8	119.09 (17)
C3—C4—H4	119.8	C12—C13—C14	119.73 (16)
C4—C5—C6	120.56 (17)	C8—C13—C14	121.17 (16)
C4—C5—H5	119.7	O2—C14—O3	122.00 (17)
C6—C5—H5	119.7	O2—C14—C13	125.66 (16)
C1—C6—C5	118.96 (16)	O3—C14—C13	112.34 (16)
C1—C6—C7	116.94 (16)	O3—C15—H15A	109.5
C5—C6—C7	124.09 (16)	O3—C15—H15B	109.5
O1—C7—N1	123.45 (17)	H15A—C15—H15B	109.5
O1—C7—C6	121.25 (16)	O3—C15—H15C	109.5
N1—C7—C6	115.29 (16)	H15A—C15—H15C	109.5
C9—C8—N1	122.04 (16)	H15B—C15—H15C	109.5
C9—C8—C13	118.94 (17)	C7—N1—C8	129.44 (16)
N1—C8—C13	119.03 (15)	C7—N1—H2	115.3
C10—C9—C8	119.93 (19)	C8—N1—H2	115.3
C10—C9—H9	120.0	C14—O3—C15	115.88 (17)

### Hydrogen-bond geometry (Å, º)

**Table:**

*D*—H···*A*	*D*—H	H···*A*	*D*···*A*	*D*—H···*A*
N1—H2···O2	0.86	1.96	2.6506 (19)	137
C1—H1···O1^i^	0.93	2.67	3.573 (2)	163
C9—H9···Cl1^i^	0.93	2.87	3.617 (2)	139

Symmetry code: (i) −*x*+2, *y*, −*z*+1/2.
